# Engineering endosomolytic nanocarriers of diverse morphologies using confined impingement jet mixing†

**DOI:** 10.1039/d3nr02874g

**Published:** 2023-09-19

**Authors:** Hayden M. Pagendarm, Payton T. Stone, Blaise R. Kimmel, Jessalyn J. Baljon, Mina H. Aziz, Lucinda E. Pastora, Lauren Hubert, Eric W. Roth, Sultan Almunif, Evan A. Scott, John T. Wilson

**Affiliations:** a Department of Biomedical Engineering, Vanderbilt University Nashville TN 37235 USA john.t.wilson@vanderbilt.edu; b Department of Chemical and Biomolecular Engineering, Vanderbilt University Nashville TN 37235 USA; c Department of Biochemistry, Vanderbilt University Nashville TN 37235 USA; d Department of Neuroscience, Vanderbilt University Nashville TN 37235 USA; e Department of Chemical Engineering, The University of Rhode Island Kingston RI 02881 USA; f NUANCE BioCryo, Northwestern University Evanston IL 60208 USA; g Department of Biomedical Engineering, Northwestern University Evanston IL 60208 USA; h Interdisciplinary Biological Sciences, Northwestern University Evanston IL 60208 USA; i Chemistry of Life Processes Institute, Northwestern University Evanston IL 60208 USA; j Simpson Querrey Institute, Northwestern University Chicago IL 60611 USA; k Robert H. Lurie Comprehensive Cancer Center, Northwestern University Chicago IL 60611 USA; l Vanderbilt Institute for Infection Immunology and Inflammation, Vanderbilt University Medical Center Nashville TN 37232 USA; m Vanderbilt Center for Immunobiology, Vanderbilt University Medical Center Nashville TN 37232 USA; n Vanderbilt Institute of Chemical Biology, Vanderbilt University Nashville TN 37235 USA; o Vanderbilt Institute of Nanoscale Science and Engineering, Vanderbilt University Nashville TN 37235 USA; p Vanderbilt-Ingram Cancer Center, Vanderbilt University Medical Center Nashville TN 37232 USA

## Abstract

The clinical translation of many biomolecular therapeutics has been hindered by undesirable pharmacokinetic (PK) properties, inadequate membrane permeability, poor endosomal escape and cytosolic delivery, and/or susceptibility to degradation. Overcoming these challenges merits the development of nanoscale drug carriers (nanocarriers) to improve the delivery of therapeutic cargo. Herein, we implement a flash nanoprecipitation (FNP) approach to produce nanocarriers of diverse vesicular morphologies by using various molecular weight PEG-*bl*-DEAEMA-*co*-BMA (PEG-DB) polymers. We demonstrated that FNP can produce uniform (PDI < 0.1) particles after 5 impingements, and that by varying the copolymer hydrophilic mass fraction, FNP enables access to a diverse variety of nanoarchitectures including micelles, unilamellar vesicles (polymersomes), and multi-compartment vesicles (MCVs). We synthesized a library of 2 kDa PEG block copolymers, with DEAEMA-*co*-BMA second block molecular weights of 3, 6, 12, 15, 20, and 30 kDa. All formulations were both pH responsive, endosomolytic, and capable of loading and cytosolically delivering small negatively charged molecules – albeit to different degrees. Using a B16.F10 melanoma model, we showcased the therapeutic potential of a lead FNP formulated PEG-DB nanocarrier, encapsulating the cyclic dinucleotide (CDN) cGAMP to activate the stimulator of interferon genes (STING) pathway in a therapeutically relevant context. Collectively, these data demonstrate that an FNP process can be used to formulate pH-responsive nanocarriers of diverse morphologies using a PEG-DB polymer system. As FNP is an industrially scalable process, these data address the critical translational challenge of producing PEG-DB nanoparticles at scale. Furthermore, the diverse morphologies produced may specialize in the delivery of distinct biomolecular cargos for other therapeutic applications, implicating the therapeutic potential of this platform in an array of disease applications.

## Abstract 2

Confined impingement jet (CIJ) mixing was utilized to fabricate pH-responsive endosomolytic polymeric nanocarriers. Manipulation of polymer and formulation properties facilitated the production of multiple nanocarriers with distinct characteristics.

## Graphical abstract

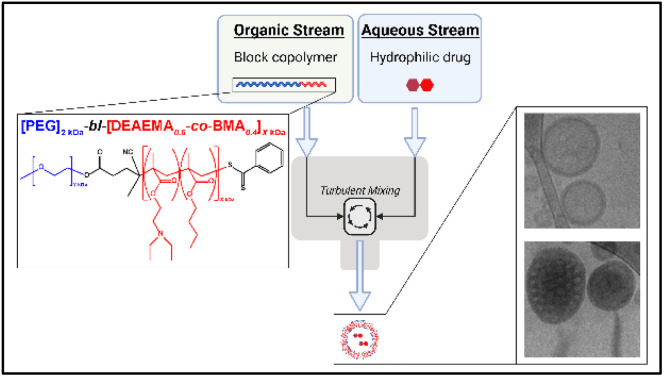

## 1. Introduction

**Fig. 1:**
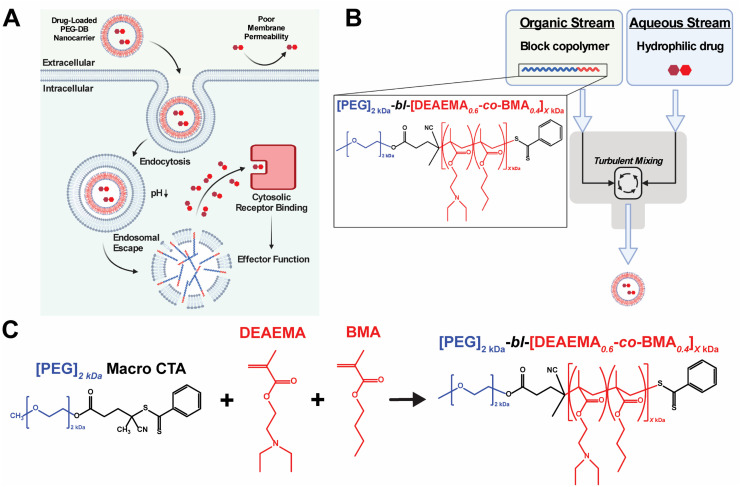
Synthesis, formulation, and evaluation of PEG-*bl*-DEAEMA-*co*-BMA nanocarriers for cytosolic drug delivery. (A) Schematic of pH-responsive PEG-DB nanocarrier facilitated cytosolic drug delivery mechanism. (B) Schematic of FNP approach to formulate PEG-DB nanocarriers. (C) RAFT polymerization scheme used to synthesize PEG-*bl*-DEAEMA-*co*-BMA diblock copolymers with varying second-block molecular weight.

Multiple classes of therapeutic biomolecules – including nucleic acids, peptides, and proteins – act on intracellular targets to affect cellular behavior and impart downstream physiological responses. Unfortunately, the efficacy and clinical translation of many biomolecular therapeutics has been limited due to the poor pharmacological properties of the native compounds, including short half-lives, rapid clearance, inadequate cellular membrane permeability, poor endosomal escape and cytosolic delivery, and susceptibility to protease/nuclease-mediated degradation.^1,2^ This challenge has inspired the development of various nanocarriers designed to improve drug pharmacokinetics (PK) and enable the cytosolic delivery of therapeutic drug cargoes.^3–5^ Many of these advances utilize pH-responsive polymers to facilitate the endosomal escape and cytosolic delivery of their therapeutic payloads.^6–13^ These systems typically exploit the pH drop from 7.4 to 5.8 that occurs during endolysosomal acidification to protonate amino groups within the polymer backbone and/or side chains. As the amines become protonated, the polymer develops a net-positive charge, stimulating interaction with the negatively charged endosomal membrane. Further, by copolymerizing hydrophobic monomers with protonatable amino monomers (p*K*_a_ 6.2–7), such polymer systems will intercalate the lipid bilayer of the endolysosome, causing endosomal membrane disruption and subsequent cytosolic delivery of loaded biomolecular cargoes^8,11,14–24^ (Fig. 1A).

A major challenge in the development of pH-responsive polymeric nanomedicines is designing nanocarriers capable of loading multiple classes of biomolecules possessing disparate physicochemical properties. Previous work in our lab has addressed this challenge *via* the implementation of multiple loading strategies including covalent conjugation, electrostatic complexation, and physical encapsulation.^11,15,21–23,25,26^ Although effective, utilizing multiple loading approaches in a single formulation can prove time and resource intensive, limiting the potential for industrial scale up and motivating the development of more scalable formulation processes. Additionally, most established formulation processes can only produce polymeric nanocarriers of a singular, defined nanoarchitecture – often micelles or polymersomes – despite evidence suggesting that nanoarchitecture influences nanocarrier transport, biodistribution, cellular uptake, targeted delivery, and drug loading.^27–31^ An ideal formulation approach would be able to produce pH responsive polymeric nanocarriers of varied nanoarchitectures in a high throughput fashion to facilitate the cytosolic delivery of diverse biomolecular cargoes to distinct cell populations.

Flash nanoprecipitation (FNP) is a scalable polymeric nanocarrier formulation technique capable of easily and reproducibly self-assembling diblock copolymers into nanocarriers of broad morphological nanoarchitectures. FNP protocols utilize a multi-inlet stream confined impingement jet (CIJ) mixer (Fig. 1B) to impinge an organic stream of hydrophobic biomolecular therapeutic and/or amphiphilic block copolymer against an aqueous stream of hydrophilic biomolecular therapeutic, forcing turbulent mixing that occurs in a matter of milliseconds.^32–35^ During the turbulent mixing process, dissolved block copolymer and biomolecule solute become both physically mixed and supersaturated, leading to phase separation that yields monodisperse polymeric nanocarriers of defined supramolecular organization and nanoarchitecture capable of encapsulating both hydrophilic and hydrophobic biomolecular cargoes.^34,36–41^ Following impingement, nanoparticles are collected in an aqueous reservoir to dilute the remaining organic solvent and to lock-in nanocarrier morphology. Early versions of FNP required the inclusion of a hydrophobic drug payload to initiate the phase separation necessary to drive particle formation.^32,33,42^ More recently, it was demonstrated that FNP could induce nanoparticle self-assembly without the incorporation of hydrophobic drug cargoes using poly(ethylene glycol)-*bl*-poly(propylene sulfide) (PEG-*bl*-PPS) diblock copolymers.^38^ This was achieved by varying the hydrophilic to hydrophobic mass fractions and molecular weights of the PEG-*bl*-PPS polymers as well as the number of impingements prior to quenching in the aqueous reservoir. Manipulation of these parameters enabled the FNP driven self-assembly of nanocarriers of diverse nanoarchitectures, including micelles, uni- and multilamellar vesicles (polymersomes), and multicompartment vesicles (MCVs) including bicontinuous nanospheres (BCNs). An added benefit of FNP is its amenability to industrial scale manufacturing. Nanocarrier platforms formulated *via* FNP can be quickly produced at scale using existing infrastructure, as evidenced by the rapid rollout of the COVID-19 mRNA vaccines produced using an industrial scale impingement jet mixing platform.^43^

Previous work in our group developed pH-responsive endosomolytic polymersomes comprised of the diblock copolymer poly(ethylene glycol)_2kDa_-*bl*-[(2-(diethylamino)ethyl methacrylate)_0.6_-*co*-(butyl methacrylate)_0.35_-*co*-(pyridyl disulfide ethyl methacrylate)_0.05_]_6kDa_ (PEG-*bl*-DEAEMA-*co*-BMA-*co*-PDSMA or PEG-DBP) capable of promoting cytosolic delivery of both small nucleic acid and peptide biomolecular cargo.^20,22^ This system actively achieves cytosolic delivery by exploiting the pH drop that occurs during endolysosomal acidification to protonate the tertiary amine on the DEAEMA monomer of the DBP block.^44^ We postulate that, following protonation, the nanoparticle disassembles and the positively charged polymer associates with the negatively charged endosomal membrane. The alkyl chain on the BMA monomer then intercalates the endosomal membrane to facilitate endosomal disruption and subsequent cytosolic release of encapsulated cargos.^6,7,14,45^ Previously, we found that PEG-DBP polymersomes enhanced the activity and pharmacological properties of cyclic dinucleotide (CDN) STING agonists to induce therapeutically relevant responses in models of melanoma, mammary carcinoma, and neuroblastoma when administered intratumorally (IT) or intravenously (IV).^20,26,46–48^ Additionally, we found that subcutaneous (SC) administration of PEG-DBP nanocarriers that co-encapsulated CDNs and peptide neoantigens invoked therapeutically relevant, antigen-specific immune responses in models of melanoma and colon adenocarcinoma.^22^ In all of these studies, we fabricated polymersomes using a modified direct hydration method in which an aqueous solution was slowly added to an ethanolic gel of PEG-DBP under sonication. While effective at loading aqueous cargo, the approach suffers from several limitations. First, the process is slow, tedious, and not amenable to scalable formulation. Second, the approach often yielded batches of nanoparticles with a high PDI (PDI > 0.2), potentially owing to the presence of other self-assembled species (*e.g.* fibrillar structures) that we sometimes observed.^22^ Third, we found that the direct hydration approach was not readily amenable to formulation of nanoparticles using copolymers with second blocks larger than ∼6 kDa, resulting in poorly defined and colloidally unstable aggregates. This prompted us to copolymerize PDSMA groups into the second DBP block to allow for covalent crosslinking of the polymersome bilayer to increase the average second block molecular weight, which results in more potent endosomal escape. However, this requires an additional crosslinking step, results in a heterogenous distribution of molecular weights, and limits the range of second block molecular weights that can be explored.

In considering both the utility and efficacy of PEG-DBP polymersomes as a platform for drug delivery as well as limitations associated with their fabrication *via* direct hydration, we investigated if FNP could formulate vesicular nanoparticles using poly(ethylene glycol)_2kDa_-*bl*-[(2-(diethylamino)ethyl methacrylate)_0.6_-*co*-(butyl methacrylate)_0.4_]_*X*kDa_ (PEG-*bl*-DEAEMA-*co*-BMA or PEG-DB) copolymers *via* the tuning of FNP parameters and PEG-DB polymer properties. We designed a library of polymers with increasing second block molecular weights and decreasing hydrophilic mass fractions (Fig. 1C and Table 1). We hypothesized that varying the hydrophilic mass fraction of the PEG-DB diblock copolymers by increasing the molecular weight of the hydrophobic DB block while maintaining a 2 kDa hydrophilic PEG block, and then formulating particles using FNP, would enable access to diverse nanoarchitectures with tunable pH-responsive, membrane-destabilizing activity. Herein, we report that, by controlling the hydrophilic mass fraction of PEG-DB polymers, the FNP process enabled the self-assembly of nanocarriers of various morphologies including micelles, unilamellar vesicles (polymersomes), and MCVs. Notably, the MCV nanoarchitecture has not been previously reported for an endosomolytic polymer such as the PEG-DB copolymer system. Using a multifaceted characterization approach, we found that impinging multiple times prior to quenching yielded nanoparticles of homogenous size and nanoarchitecture. Furthermore, we demonstrated that regardless of polymer length, particle size, and morphology, nanocarriers formulated *via* FNP remained pH-responsive. Finally, when loaded with 2′,3′ cyclic guanosine monophosphate–adenosine monophosphate (cGAMP), FNP-formulated nanocarriers were therapeutically active in a murine model of melanoma. Collectively, these results demonstrate that the tuning of PEG-DB copolymer composition and FNP parameters reproducibly yields nanoparticles of multiple, diverse morphological nanoarchitectures to deliver various classes of biomolecular drug cargoes, thus establishing a nanocarrier platform with a wide range of potential therapeutic applications.

**Table 1:** Properties of PEG_2kDa_-*bl*-(DEAEMA-*co*-BMA)_*X*kDa_ polymers

Polymer name	DP	% DEAEMA	% BMA	2^nd^ block MW (kDa)	Hydrophilic block weight fraction (*f*)	Polymer MW (kDa)
3	20.3	89.3	10.7	3.67	0.382	5.93
6	39.9	61.9	38.1	6.74	0.251	9.00
12	76.5	61.4	38.6	12.90	0.149	15.17
15	92.0	62.6	37.4	15.57	0.127	17.83
20	118.1	62.3	37.7	19.96	0.102	22.22
30	155.1	61.1	38.8	26.14	0.080	28.40

## 2. Results and discussion

### 2.1. Flash nanoprecipitation produces homogenous nanoparticles of various sizes and morphologies dependent on the hydrophilic block molecular weight fraction

Polymers were synthesized according to procedures reported previously.^20^ In short, reversible addition–fragmentation chain transfer (RAFT) polymerization was used to synthesize a series of pH-responsive poly(ethylene glycol)_2kDa_-*bl*-[(2-diethylaminoethyl methacrylate)_0.6_-*co*-(butyl methacrylate)_0.4_]_*X*kDa_ (PEG-DB) diblock copolymers (Fig. 1C). In this work, we primarily sought to examine the effect of varying the weight fraction of the copolymer second block on the properties of resultant nanocarriers formulated *via* FNP. We held the hydrophilic PEG first block constant at 2 kDa and varied the hydrophobic DB second block molecular weight from 3 kDa to 30 kDa. The complete library of polymers and their properties is detailed in Table 1.

**Fig. 2:**
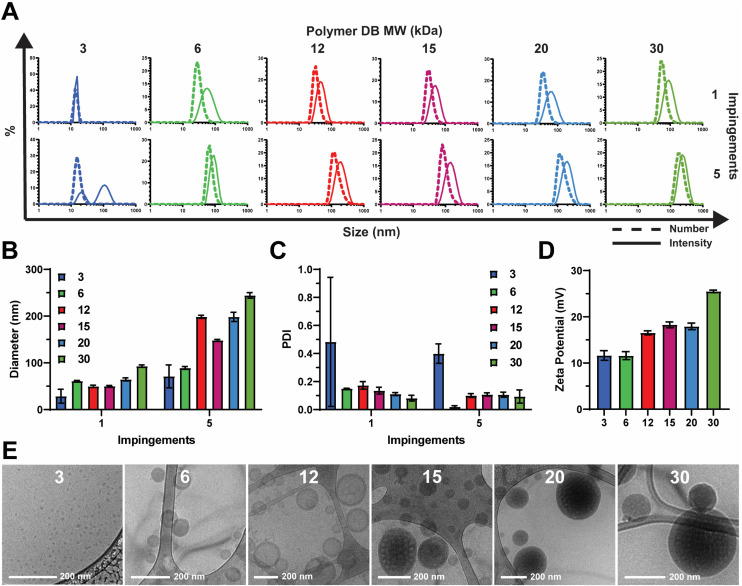
Effect of hydrophilic block weight fraction on nanocarriers formulated *via* FNP. (A) Size distributions, (B) intensity average diameter, and (C) PDI of indicated nanocarriers formulated using 1 or 5 impingements. (D) Zeta potential and (E) representative cryogenic transmission electron microscopy (cryoTEM) images of indicated nanocarriers.

We employed a flash nanoprecipitation process to formulate the PEG-DB nanoparticles. First, we varied the number of impingements of solubilized polymer in a 50 : 50 solvent : antisolvent mixture within the CIJ mixer prior to quenching in excess DI H_2_O. Previous reports demonstrate that impingement number influences sample polydispersity (PDI) and size, while producing various nanoparticle morphologies including polymersomes, micelles, filomicelles, and multicompartmental vesicles.^38^ Increasing the number of impingements prior to quenching generally caused an increase in nanoparticle hydrodynamic diameter and a decrease in polydispersity index (PDI) as measured by DLS (Fig. 2A–C). For both the 1 and 5 impingement formulations, particle size generally trended upward with decreasing hydrophilic block molecular weight fraction (Fig. 2B and C). This was reflected by a noticeable increase in turbidity of the formulations corresponding to decreasing hydrophilic block molecular weight fraction (Fig. S2†). As we desired a highly homogenous nanoparticle formulation in our drug carrier to ensure reproducible performance, we opted to use 5 impingements in all successive studies unless otherwise indicated. We found that the surface charge of the resultant nanoparticles was slightly cationic and positively correlated with increasing DB block molecular weight (Fig. 2D). These data suggest that because PEG molecular weight was fixed at 2 kDa, the degree of PEG corona shielding decreased as DB block molecular weight increased, due to the exposure of additional cationic DEAEMA groups on the nanoparticle surface.

To analyze the morphological nanoarchitecture of the resultant nanoparticles, nanoparticles formulated from each PEG-DB polymer were imaged *via* cryogenic transmission electron microscopy (cryoTEM). CryoTEM images indicated DB3 kDa formed micelles, DB6 kDa and DB12 kDa formed single-compartment vesicles (polymersomes), DB15 kDa formed a mixture of single- and multi-compartment vesicles (MCVs), and DB20 kDa and DB30 kDa formed MCVs (Fig. 2E). This suggests that there is a critical second block molecular weight between 12 and 15 kDa that is required to form a MCV nanoarchitecture, and we hypothesize that this morphological phase change may explain why DB15 kDa formed particles with a smaller average diameter than DB12 kDa after 5 impingements (Fig. 2A and B). Additionally, for all samples, apparent particle sizes observed *via* cryoTEM agreed with what was reported by DLS for both the 1 and 5 impingement formulations (Fig. 2A, B, E and S3†). To further confirm nanoparticle morphology, nanoparticles formulated from DB3 kDa, DB6 kDa, and DB12 kDa polymers were analyzed *via* small angle X-ray scattering (SAXS) and fitted to models of micellar or vesicular morphology, respectively. It was determined that DB3 kDa nanoparticles exhibited a micelle nanoarchitecture, and that DB6 kDa and DB12 kDa nanoparticles exhibited a vesicle nanoarchitecture (Fig. S6†). In concert, these data demonstrate that FNP can formulate nanoparticles of various nanoarchitectures using a pH-responsive PEG-DB copolymer, and that nanoarchitecture is dependent on the molecular weight fraction of the hydrophilic PEG first block.

### 2.2. Nanocarriers exhibit pH-sensitive and membrane lytic activities

**Fig. 3:**
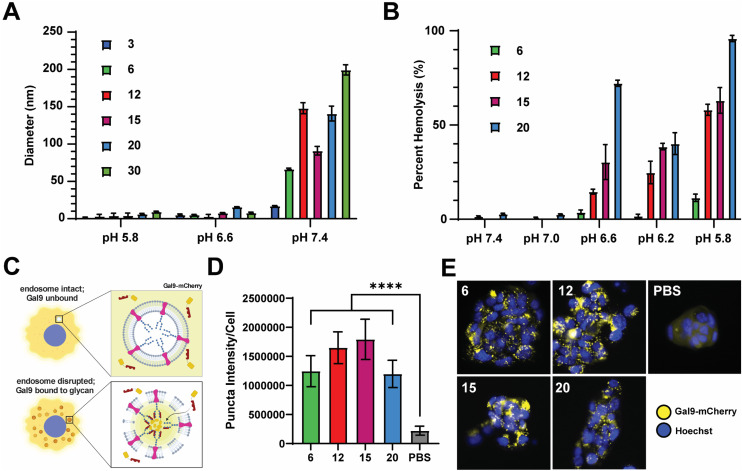
PEG-*bl*-DEAEMA-*co*-BMA nanocarriers formulated *via* FNP exhibit pH responsive and membrane-destabilizing properties. (A) Number average diameter of indicated nanocarrier after incubation in PBS at indicated pH. (B) Relative degree of erythrocyte hemolysis of indicated nanocarriers at indicated pH values. (C) Schematic depicting Gal9–mCherry assay used to investigate the degree of endosomal disruption of nanocarriers. (D) Integrated pixel intensity of the Gal9–mCherry puncta per cell for indicated nanocarriers (mean ± SEM, *n* = 16). Analyzed *via* one-way ANOVA with Tukey's *post-hoc*. ****P < 0.0001. (E) Representative images of NCI-H358 cells expressing Gal9-YFP following overnight treatment with indicated nanocarriers displaying distinct puncta formation.

We next sought to evaluate the relative pH-responsive membrane-disruptive capacity of each nanocarrier, a property of PEG-DB polymers that tends to correlate with efficiency of endosomal escape of associated cargoes to the cytosol. To demonstrate pH-responsive disassembly, nanoparticles were suspended in PBS buffers at pH 7.4 (physiological), pH 6.6 (early endosome), and pH 5.8 (late endosome), incubated for 1 hour at 37 °C, and subsequently measured for size. DLS measurements indicated that the all PEG-DB nanocarriers disassembled in response to physiological acidic environmental conditions (Fig. 3A). We then employed an erythrocyte hemolysis assay, in which deidentified human erythrocytes were incubated with nanocarriers at physiological temperature (37 °C) and at a pH range consistent with that exhibited during endolysosomal acidification, to evaluate pH-dependent membrane-lytic capability. Hemoglobin leakage, indicative of cell lysis, was quantified after incubation using absorbance spectroscopy, and it was determined membrane destabilization positively correlated with increasing DB block molecular weight and negatively correlated with increasing pH (Fig. 3B). These results were anticipated, as carriers with larger second block molecular weight fractions possess longer protonated DEAEMA chains and exposed membrane-lytic domains within the late endosome to facilitate increased membrane disruption. Minimal hemolysis prevalent at pH 7.4 (physiological) for all carriers indicated structural stability outside of the endosome, highlighting the ability of these delivery vehicles to specifically delivery cargo intracellularly without undesired premature disassembly and release.

We next employed a galectin recruitment assay to demonstrate the ability of each formulation to induce endosomal rupture. As reported previously, NCI-H358 cells were engineered to express a fusion protein of galectin 9 and mCherry (Gal9–mCherry).^49^ Following disruption of intracellular endosomes, intraluminal glycans previously shielded by the endosomal membrane become exposed to the cytosol. Gal9, typically diffuse throughout the cytosol, is recruited to the site of the ruptured endosome to bind the newly exposed glycans. By fusing Gal9 to a fluorescent protein such as mCherry, endosomal rupture can be visualized by the redistribution of fluorescent signal from the cytosol to distinct puncta at the sites of ruptured endosomes (Fig. 3C). NCI-H358 cells stably expressing Gal9–mCherry were incubated with nanocarrier formulations for 18 h prior to imaging. Using an image processing algorithm that quantifies the integrated pixel intensity of each punctate spot normalized to the cell count per image, representative of the mCherry redistribution that occurs following endosomolysis on a per cell basis, it was determined that all formulations tested, DB6 kDa, DB12 kDa, DB20 kDa, and DB30 kDa, could induce significant endosomal disruption compared to a saline treated control (Fig. 3D). Representative images of NCI-H358 cells 18 h after treatment with indicated nanocarriers all depict puncta formation consistent with endosomal rupture (Fig. 3E). Collectively, these data indicate that all tested PEG-DB nanocarriers are pH responsive and capable of successfully inducing endosomolysis to facilitate the cytosolic delivery of biomolecular cargo to recipient cells.

### 2.3. Nanocarriers efficiently load and deliver relevant biomolecular cargoes intracellularly

**Fig. 4:**
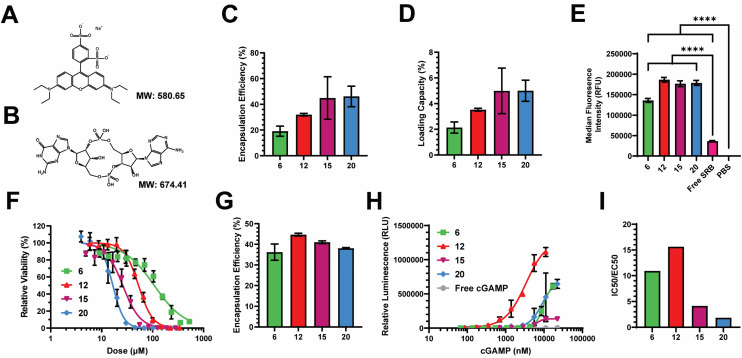
Evaluation of the abilities of PEG-*bl*-DEAEMA-*co*-BMA nanocarriers to encapsulate therapeutic and model compounds and facilitate their intracellular delivery. Structure and molecular weight of (A) sulforhodamine-B (SRB) and (B) 2′,3′ cyclic guanosine monophosphate–adenosine monophosphate (cGAMP). (C) Encapsulation efficiency and (D) loading capacity of SRB within indicated nanocarriers. (E) MFI of MDA-MB-231 cells treated with equivalent doses of SRB as a free compound or within indicated nanocarriers overnight (mean ± SEM, *n* = 3–6). Analyzed *via* one-way ANOVA with Tukey's *post-hoc*. ****P < 0.0001. (F) Cytotoxicity curves of empty nanocarriers in A549 cells. (G) Encapsulation efficiency of cGAMP within indicated nanocarriers. (H) Dose response curves of STING activation following treatment with indicated cGAMP concentrations encapsulated within indicated nanocarriers. (I) Ratio of IC50 : EC50 to reflect the abilities of indicated nanocarriers to activate the STING pathway with minimal polymer-induced cytotoxicity.

We next assessed the loading efficiency of each nanocarrier using both model and therapeutic compounds. As a model compound, we examined the loading efficiency of sulforhodamine B (SRB) – a negatively charged, hydrophilic, small molecule, fluorescent dye. We selected SRB as it has a similar molecular weight and charge to cGAMP (Fig. 4A and B), which is an innate immune agonist of the stimulator of interferon genes (STING) that we have previously loaded into PEG-DB nanocarriers.^20,22,26,46–48^ After impinging each polymer at 10 mg mL^−1^ in the organic stream five times against SRB dissolved at 1 mg mL^−1^ in the aqueous stream, unencapsulated SRB was removed *via* dialysis and SRB encapsulation was quantified *via* fluorescence spectroscopy. The encapsulation efficiency (EE) trended slightly upward with increasing second block MW, maintaining between 20–50% depending on polymer (Fig. 4C). The loading capacity (LC) trended similarly, ranging between 2–5% depending on the polymer (Fig. 4D). We then evaluated the ability of PEG-DB nanocarriers to facilitate the cellular uptake of SRB. MDA-MB-231 human breast cancer epithelial cells were treated with 20 μg mL^−1^ of SRB within indicated nanocarriers or as a free molecule overnight. Cells were then collected, washed, and analyzed *via* flow cytometry to assess SRB uptake. Compared to both free SRB and PBS treated controls, all nanocarriers were all able to significantly increase the cellular uptake of SRB without inducing cytotoxicity, suggesting that cells more readily internalize the small molecule when it is encapsulated within a PEG-DB nanocarrier (Fig. 4E and S5†).

Prior to conducting therapeutic studies, we evaluated the relative toxicity of each polymeric nanocarrier absent any loaded cargoes. A549 human lung adenocarcinoma cells were incubated with increasing concentrations of each nanoparticle. Cell viability following incubation indicated that cytotoxicity increased with decreasing hydrophilic block molecular weight fraction, as equivalent cytotoxicity values could be achieved with lower concentrations of polymers with smaller hydrophilic block molecular weight fractions (Fig. 4F). This is likely a combined effect of the large DB block length and cationic surface charge, as highly cationic particles are more likely to interact with and damage cell membranes, inducing cytotoxicity.^50–54^

Given the similar physicochemical properties between SRB and cGAMP, these data suggested to us that PEG-DB nanocarriers formulated *via* FNP may be suitable for the cytosolic delivery of cGAMP, a potent agonist and endogenous ligand of the cGAS-STING pathway. Activation of STING triggers a type I interferon (IFN-I)-driven inflammatory response that stimulates dendritic cells to cross-present antigen on class I major histocompatibility complex (MHC). This pathway can be exploited to cross-present tumor antigens to prime antitumor CD8^+^ cytotoxic T cells, allowing for the reprogramming of the TME to an immunogenic, tumoricidal phenotype necessary for effective tumor growth inhibition.^55,56^ Each polymer was impinged five times at 10 mg mL^−1^ in the organic stream against a 0.5 mg mL^−1^ cGAMP solution in the aqueous stream. Particles were purified *via* dialysis and cGAMP encapsulation was quantified *via* high performance liquid chromatography (HPLC). The EE of cGAMP was determined to be between 35–45% for all formulations (Fig. 4G). To evaluate the relative activity of each formulation, THP1-Dual ISG Cells were used. These cells secrete luciferase following IFN-I transcriptional activation, enabling the quantification of IFN-I activation *via* luminescence spectroscopy (Fig. 4H). Using this assay, we were able to determine that all formulations enhanced the activity of cGAMP, with EC50 values of 9.9, 3.4, 6.3, and 8.9 μM, respectively, for the DB6 kDa, DB12 kDa, DB15 kDa, and DB20 kDa nanoparticles. We quantified the ratio of IC50, determined by relative cytotoxicity, to EC50, determined by THP-1 IFN-I activation, to evaluate a lead nanocarrier formulation(s) for cGAMP delivery (Fig. 4I). Higher IC50 : EC50 ratios indicate that the formulation has a more desirable potency to toxicity profile. DB6 kDa and DB12 kDa nanocarriers had IC50 : EC50 ratios approximately 2–5 times greater than the DB15 kDa and DB20 kDa nanocarriers, indicating that DB6 kDa and DB12 kDa polymers generate superior cGAMP delivery vectors. This is logical as DB6 kDa and DB12 kDa polymers created polymersomes with aqueous cores for the physical encapsulation of cGAMP (Fig. 2E and S6†), while containing lower molecular weight fractions of the hydrophobic, cationic DB block that mediates membrane-lytic cytotoxicity.

### 2.4. cGAMP-loaded PEG-DB nanocarriers formulated *via* FNP improve therapeutic cGAMP efficacy in a murine melanoma model

To evaluate the performance of pH responsive PEG-DB nanocarriers formulated *via* FNP in a therapeutic context, we formulated cGAMP-loaded PEG-DB nanoparticles by impinging an organic solution of 10 mg mL^−1^ DB6 kDa polymer five times against an aqueous solution of 0.5 mg mL^−1^ cGAMP. These STING-activating nanoparticles (STANs) encapsulated cGAMP to facilitate its cellular uptake and subsequent cytosolic delivery. Activation of the STING pathway within the tumor microenvironment (TME) polarizes tumor associated macrophages (TAMs) and other resident antigen presenting cells (APCs) towards a pro-inflammatory phenotype. In turn, this facilitates the activation and expansion of tumor antigen-specific T cells, the recruitment of effector T cells to the TME, and the T cell mediated killing of tumor cells.^55–58^ Furthermore, we previously demonstrated that systemic administration of STANs normalizes the tumor vasculature to further enhance T cell infiltration and improve antitumoral responses in multiple murine cancer models.^26^

**Fig. 5:**
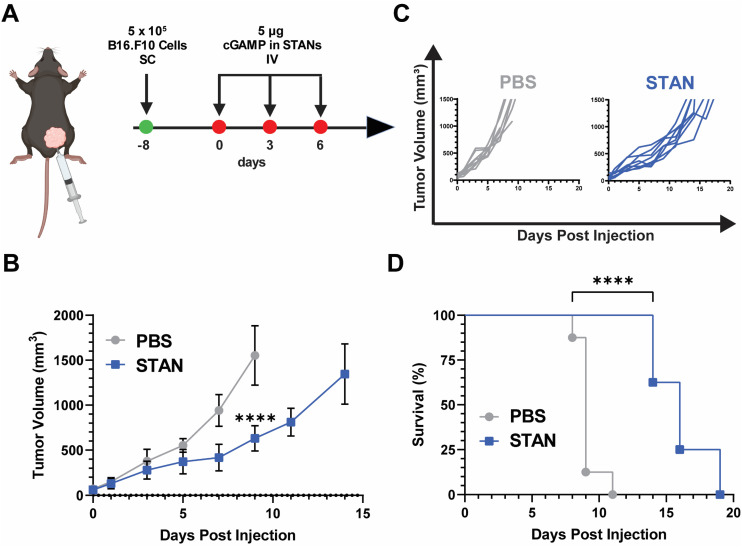
*In vivo* efficacy of STANs formulated *via* FNP in a murine B16.F10 model of melanoma. (A) Treatment timeline of B16.F10 tumor model. (B) Mean tumor volume of mice treated with 5 μg of cGAMP within STANs plotted until the death of the first mouse within each group. Statistical analysis was performed on day 9 when the first mouse of any treatment group died (mean ± SEM, *n* = 6–8). Analyzed *via* Student's two-tailed *t*-test. ****P < 0.0001. (C) Corresponding spider curves of mice treated with PBS or STANs. (D) Kaplan–Meier survival curves of mice treated with PBS or STANs (*n* = 8). Analyzed *via* Mantel–Cox log-rank test. ****P < 0.0001.

The DB6 kDa polymer was selected as it had a favorable IC50 : EC50 ratio (Fig. 4C), possessed the same second block molecular weight and mass fraction as our previously reported STAN platforms,^20,22^ and its approximately 100 nm size suggested that it may be able to exploit the enhanced permeation and retention (EPR) effect of solid tumors.^59^ On Day 8, 5 × 10^5^ B16.F10 cells were inoculated SC in the right flank. Injections began on Day 0, when average tumor volume reached 50 mm^3^. Mice were administered 5 μg of cGAMP encapsulated within STANs IV on days 0, 3, and 6 (Fig. 5A). Tumor volumes were measured every other day and mice were sacrificed when tumor volume reached 1500 mm^3^. The 5 μg STAN dose significantly inhibited tumor growth compared to a PBS treated control (Fig. 5B). Additionally, STAN treatment significantly extended survival compared to PBS (Fig. 5D). A free cGAMP control group was not tested as we have previously demonstrated that free cGAMP does not improve therapeutic responses to B16.F10 melanoma when administered IV.^20^ Notably, our previous formulation approach necessitated the IV administration of 10 μg of cGAMP within STANs to observe therapeutically significant results,^20,26,46–48^ suggesting that STANs formulated *via* FNP may be more potent than our previous platform. This observation warrants further investigation.

In concert, these results demonstrate that STANs formulated *via* FNP inhibit tumor growth and extend survival in a B16.F10 melanoma model. Moreover, these data showcase the ability of PEG-*bl*-DEAEMA-*co*-BMA nanocarriers formulated *via* FNP to improve cGAMP delivery *in vivo* following IV administration, indicating that FNP may be a viable alternative formulation approach for our previously characterized STANs,^20,26,47^ especially given that STANs formulated *via* FNP can suppress B16.F10 tumor growth using half of the dose of our previous work. As the purpose of these studies was to test nanocarrier activity in an *in vivo* context, as opposed to optimize a nanocarrier for IV cGAMP administration, further studies are needed to evaluate if other carrier formulations are advantageous for IV cGAMP immunotherapy applications. Additionally, as cGAMP was selected as a model therapeutic because it is a cytosolically-acting compound that our lab has previous experience in designing carrier platforms for,^20,24^ the ability of different PEG-DB nanocarriers formulated *via* FNP to encapsulate and deliver other physicochemically distinct biomolecules with therapeutic relevance to various diseases warrants further investigation.

## 3. Conclusions

In this work, we optimized an FNP approach to formulate PEG-DB nanocarriers of diverse morphological nanoarchitectures without the necessity for hydrophobic drug incorporation to drive nanoparticle assembly. By varying the hydrophilic mass fraction, our approach was able to formulate homogenous populations of nanoparticles with low polydispersities, that possessed various morphologies, including micelles, polymersomes, and MCVs. These results evidenced the ability to harness FNP to formulate endosomolytic polymer nanocarriers for delivery of therapeutically promising drug payloads to hard-to-access cytosolic targets. As these studies only examined the therapeutic delivery of cGAMP, a hydrophilic small molecule that packages easily into aqueous cores, additional studies examining the therapeutic applications of FNP formulated PEG-DB nanocarriers encapsulating other therapeutic compounds merit investigation. Nevertheless, these investigations establish FNP as an effective formulation approach for PEG-DB nanocarriers, including as a carrier for increasing the efficacy of the immunotherapeutic cGAMP, and demonstrate that FNP can produce PEG-DB nanocarriers of an array of morphologies, which may offer unique advantages for the loading and delivery of other molecules.

## 4. Materials and methods

### 4.1. Materials

All materials were purchased from Sigma-Aldrich unless otherwise specified.

### 4.2. Methods

#### 4.2.1. Polymer synthesis

The reversible addition–fragmentation chain transfer (RAFT) polymer synthesis method utilized in this experiment has been described previously.^20^ Briefly, the monomers, butyl methacrylate (BMA) and 2-(diethylamino) ethyl methacrylate (DEAEMA) (Tokyo Chemical Industry) were reacted with poly(ethylene glycol) 4-cyano-4-(phenylcarbonothioylthio)pentanoate (PEG CTA) and 4-4′-AZO-bis(4-cyanovaleric acid) initiator (V501) (MP Biomedicals) for 18 h at 70 °C in a reaction vessel following a 30 min purge with gaseous nitrogen. Polymers were then purified *via* dialysis through an acetone-deionized water (DI H_2_O) gradient for 48 h in 3500 Da MWCO SnakeSkin dialysis tubing (Thermo Scientific), and subsequently lyophilized for 48 h in a FreeZone Benchtop Freeze Dry System (Labconco). ^1^H-NMR spectroscopy in CdCl_3_ was analyzed pre- and post-reaction and after lyophilization using a 400 MHz NMR Spectrometer (Bruker), courtesy of Vanderbilt University's Small Molecule NMR Facility Core, to determine conversion and composition, respectively (Fig. S1†).

#### 4.2.2. Nanoparticle formulation using confined impingement jet mixing

Nanoparticles were formulated using a multi-inlet stream confined impingement jet (CIJ) mixer (Holland Applied Technologies). For each sample, the indicated polymer was dissolved at 10 mg mL^−1^ in tetrahydrofuran (THF) and 1 mL was aspirated into a disposable 1 mL polypropylene syringe (Fisher). A second syringe was used to aspirate 1 mL of an aqueous solution containing either DI H_2_O alone for empty nanoparticles or solubilized hydrophilic drug/drug analogue. The syringes were connected to the inlets of the CIJ unit, and then rapidly depressed simultaneously in less than 0.5 s to induce turbulent mixing within the chamber, as previously reported.^60^ For each impingement, the resulting mixture was collected in a 20 mL scintillation vial, and the total volume was split evenly between the two syringes. The impingement process was repeated four times for a total of five impingements per batch of fabricated nanoparticles. On the final impingement, the mixture was collected in an aqueous quench bath of 2× the remaining syringe volume of DI H_2_O, while undergoing vigorous stirring, to dilute the THF cosolvent to a degree capable of inducing chain stabilization and establishing the morphology of the nanoparticles. Particles were purified *via* dialysis in 3500 Da SnakeSkin Dialysis Tubing (Thermo Scientific) for 48 h against DI H_2_O to remove residual THF prior to downstream experimentation.

#### 4.2.3. Size, PDI, and zeta potential measurements

Size, PDI, and zeta potential values of nanoparticle samples were analyzed using a Zetasizer Advance Range (Malvern Panalytical), courtesy of the Vanderbilt Institute for Nanoscale Science and Engineering. For size and PDI measurements, purified sample was diluted 10× in 0.22 μm sterile-filtered phosphate-buffered saline (PBS) (Gibco) in a 1.5 mL semi-micro cuvette (Fisher) prior to analysis to mimic physiological osmolarity and pH. For zeta potential measurements, purified sample was diluted 10× in 0.22 μm sterile-filtered 11.1 mM NaCl (for a final concentration of 10 mM NaCl), and transferred to a DTS1070 capillary cell for measurement.

#### 4.2.4. Small-angle X-ray scattering (SAXS) analysis

SAXS measurements were conducted at the 5-ID beamline of the DuPont-Northwestern-Dow Collaborative Access Team (DND-CAT), located at the Advanced Photon Source in Argonne National Laboratories. The experiments utilized collimated X-rays with a wavelength (*λ*) of 1.24 Å and an energy source of 9 keV. All samples, prepared at a concentration of 1 mg mL^−1^, were analyzed using an in-vacuum flow cell system within quartzy capillaries measuring 1.6 mm in thickness. The scattering data were collected in the q-range of 0.0015 to 0.08 Å^−1^, with a sample-to-detector distance of approximately 8.5 m and an exposure time of 5 s. The beamline was calibrated using silver behenate and a gold-coated silicon with a grating density of 7200 lines per mm. The momentum transfer vector *q* is defined as *q* = (4π/*λ*)sin *θ*, where 2*θ* is the scattering angle. Data reduction and buffer subtraction were performed using BioXTAS RAW software,^61^ while model fitting was carried out using the SasView 5.0.5 software package.

#### 4.2.5. Cryogenic transmission electron microscope (cryoTEM) imaging of nanoparticles

Prior to plunge-freezing, 200 mesh Cu grids with a lacey carbon membrane (EMS Cat# LC200-CU-100) were glow-discharged in a Pelco easiGlow glow discharger (Ted Pella Inc., Redding, CA, USA) using an atmosphere plasma generated at 15 mA for 15 seconds with a pressure of 0.24 mbar. This treatment created a negative charge on the carbon membrane, allowing aqueous samples to spread evenly over of the grid. 4 μL of sample was pipetted onto the grid and blotted for 5 seconds with a blotting pressure of 1, followed by immediate plunging into liquid ethane within an FEI Vitrobot Mark IV plunge freezing instrument (Thermo Fisher Scientific, Waltham, MA, USA). Grids were then transferred to liquid nitrogen for storage. For the 5 impingement samples, the plunge-frozen grids were kept vitreous at −180 °C in a Gatan ELSA cryo transfer holder (Gatan Inc., Pleasanton, CA, USA) while viewing in a JEOL JEM1400 LaB_6_ emission TEM (JEOL USA, Inc., Peabody, MA) at 120 keV. Image data was collected by a Gatan OneView camera (Gatan Inc., Pleasanton, CA, USA). For the 1 impingement samples, the plunge-frozen grids were kept vitreous at −180 °C in a Gatan cryo transfer holder model 626.5 (Gatan Inc., Pleasanton, CA, USA) while viewing in a JEOL JEM1230 tungsten emission TEM (JEOL USA, Inc., Peabody, MA,) at 120 keV. Image data was collected by a Gatan Orius SC1000 CCD camera Model 831 (Gatan Inc., Pleasanton, CA, USA).

#### 4.2.6. Erythrocyte hemolysis assay

A Genesys-150 ultraviolet-visible (UV/vis) spectrophotometer (Thermo Scientific) was used to determine exact polymer concentrations in nanoparticle samples after formulation *via* comparison of the absorbance of the sample at 310 nm (a characteristic peak of the macro CTA) to that of standards of known polymer concentration. Nanoparticles were then diluted to concentrations of 10, 5, and 1 μg mL^−1^. 7 μL of each formulation was seeded into a 96-well V-bottom plate (Greiner Bio-One). 0.1% Triton X-100 and 1× PBS were used as positive and negative controls, respectively. De-identified human whole blood, obtained courtesy of the Vanderbilt Hematology Core, was washed by centrifuging at 450*g* for 5 min, aspirating off the plasma layer, resuspending in 1× PBS, and repeating the washing procedure four times. The resulting hematocrit was then diluted 50× in PBS at various pH-values associated with endolysosomal trafficking (pH 5.8, 6.2, 6.6, 7.0, and 7.4). 168 μL of diluted blood was added to each nanoparticle formulation or control in the 96-well V-bottom plate, and suspensions were incubated at 37 °C for 1 h. Plates were then centrifuged at 600*g* for 1 min, and 80 μL of supernatant was collected and transferred to a clear, 96-well flat bottom plate (Greiner Bio-One). The amount of hemoglobin leakage into the supernatant was determined *via* absorbance spectroscopy (Synergy H1, *λ* = 575 nm), and percent hemolysis was calculated as the ratio of (Abs_Sample_ − Abs_PBS_)/(Abs_Triton_ – Abs_PBS_).

#### 4.2.7. pH-Dependent size transition

Following formulation, indicated nanoparticles were diluted 10× into PBS at pH values of 7.4 (physiological), 6.6 (early endosomal), and 5.8 (late endosomal). Nanoparticles were incubated in each buffer for 1 h at 37 °C. Following incubation, samples were transferred to 1.5 mL semi-micro cuvettes (Fisher) and analyzed *via* DLS using a Zetasizer Advance Range (Malvern Panalytical) courtesy of the Vanderbilt Institute of Nanoscale Science and Engineering.

#### 4.2.8. Gal9–mCherry assay

Gal9 recruitment assays were performed as previously described^49^ with minor modifications as follows. NCI-H358 cells stably expressing Gal9–mCherry were seeded in 96-well black walled clear bottom plates (Grenier, catalog number 655090) at a density of 5000 cells per well and allowed to adhere overnight. The following day, cells were treated with nanoparticle formulations at indicated concentrations and incubated for an additional 15 hours. Media was then exchanged with 100 μL of imaging media (FluoroBrite DMEM supplemented with 25 mM HEPES, 10% FBS, 1% Pen/Strep, and 4 μM Hoechst 33342). Cells were imaged using an ImageXpress Nano Automated Imaging System (Molecular Devices) with a 20× Nikon CFI60 series objective, courtesy of the Vanderbilt High Throughput Screening Core. Images were analyzed using the Transfluor Application Module within the MetaXpress Software (Molecular Devices), which blindly counted the integrated pixel intensity of the Gal9 + spots within each image.

#### 4.2.9. Cell culture

A549 human alveolar basal epithelial adenocarcinoma cells (Invivogen) and MDA-MB-231 human breast cancer epithelial cells were maintained in Dulbecco's modified Eagle's medium (Gibco) supplemented with 10% fetal bovine serum (Invitrogen, Carlsbad, CA), 100 U mL^−1^ penicillin (Gibco), 100 μg mL^−1^ streptomycin (Gibco). NCI-H358 epithelial human lung cancer cells engineered to express a Gal9–mChery fusion (NCI-H358-Gal9–mCherry) were generously provided by collaborators at AstraZeneca.^49^ NCI-H358 cells were maintained in RPMI-1640 medium (Gibco) supplemented with 10% FBS (Gibco), 100 U mL^−1^ penicillin (Gibco), 100 μg mL^−1^ streptomycin (Gibco), and 1× GlutaMAX (Gibco). THP1-Dual ISG monocytes (Invivogen) were maintained in RPMI 1640 supplemented with 2 mM l-glutamine, 25 mM HEPES, 10% FBS (Gibco), 100 U mL^−1^ penicillin (Gibco), 100 μg mL^−1^ streptomycin (Gibco), and 100 μg mL^−1^ Normocin (Invivogen). 100 μg mL^−1^ zeocin and 10 μg mL^−1^ blasticidin were added every other passage to maintain selection pressure. Cells were passaged when confluency reached 70–90% using 0.05% trypsin (Gibco).

#### 4.2.10. Cell viability

Cell viability was measured using a CellTiter-Glo Luminescent Cell Viability Assay (Promega). Briefly, A549 adenocarcinomic human alveolar basal epithelial cells were seeded at a density of 5000 cells per well in a clear-bottom, white-walled 96-well plate (Greiner Bio-One) and treated with empty nanoparticle formulations for 24 hours. CellTiter-Glo reagent was added per the manufacturer's instructions, and the plate was allowed to incubate for 30 min before measuring luminescence using a Synergy HI plate reader (Bio-Tek). Relative viability was calculated by normalizing luminescence readings to a control group of cells treated with PBS. IC_50_ values were extrapolated by performing a nonlinear regression curve fit on the cytotoxicity data points using Prism 9.5.1 (GraphPad) software.

#### 4.2.11. Relative cGAMP activity

An aqueous stream of 0.5 mg mL^−1^ cGAMP impinged five times against an organic stream of 10 mg mL^−1^ of indicated polymer to form several STAN formulations. cGAMP activity was measured using THP1-Dual ISG Cells (Invivogen). Cells were seeded in a 96-well plate at a density of 20 000 cells per well and allowed to adhere overnight. The following day, cells were treated with indicated concentrations cGAMP within STAN formulations or as a free drug for 24 h. Relative expression of IFN-I was measured using Quanti-Luc reagent (InvivoGen) using a Synergy HI plate reader (Bio-Tek) per the manufacturer's instructions.

#### 4.2.12. Quantification of SRB EE and LC

SRB-loaded nanocarriers were formulated *via* impinging an aqueous stream containing 1 mg mL^−1^ SRB five times against an organic stream containing 10 mg mL^−1^ of indicated polymer. Unencapsulated SRB was removed from the formulation *via* dialysis against DI H_2_O for 48 h in 3500 Da MWCO SnakeSkin Dialysis Tubing (Thermo Scientific). DI H_2_O was exchanged approximately every 8 h during this process.

Purified samples were diluted 2× in ethanol to disrupt the nanoarchitecture and release encapsulated drug, diluted to a measurable concentration, and analyzed for fluorescent intensity (Ex/Em: 565/586 nm) using a Synergy-H1 plate reader (Bio-Tek). Fluorescence readouts were normalized to a blank, and model drug concentrations were determined by use of a standard curve of fluorescence of known SRB concentrations. Encapsulation efficiency (EE) was calculated as the ratio of the mass of drug encapsulated in the purified sample to the total mass of drug added into the system, as per the following equation:
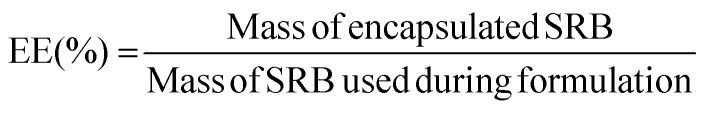


Loading capacity (LC) was calculated as the ratio of the mass of drug encapsulated in the purified sample to the mass of polymer in the sample, as per the following equation:
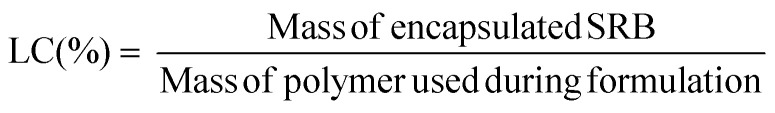


#### 4.2.13. Cellular uptake of SRB-loaded nanocarriers

SRB-loaded nanoparticles were formulated by impinging an organic stream of 10 mg mL^−1^ of indicated polymer dissolved in THF against an aqueous stream of 1 mg mL^−1^ SRB dissolved in DI H_2_O. The formulation was quenched during the fifth impingement by impinging into a reservoir of 2× excess of DI H_2_O relative to the volume remaining in the syringes while subjected to rapid stirring. Unencapsulated SRB was removed *via* dialysis against DI H_2_O in 3500 Da MWCO SnakeSkin dialysis tubing (Thermo Scientific) for 48 h with periodic water changes. After purification, SRB encapsulation was quantified *via* fluorescent spectroscopy as described previously.

MDA-MB-231 breast cancer cells were seeded in 12-well plates at a density of 200 000 cells per well and incubated overnight to permit adherence to the plate bottom. The following day, cells were treated with 20 μg mL^−1^ of SRB administered either freely or within the indicated nanocarrier. Cells were incubated with SRB formulations overnight. On the third day, cells were collected *via* trypsinization, washed, stained for viability with 1 μg mL^−1^ DAPI, and analyzed using a CellStream flow cytometer (Cytek). Events were sequentially gated for Cells, Single Cells, and then Live Cells (Fig. S4†). Within the Live Cells gate, the median fluorescence intensity (MFI) for the SRB channel was plotted.

#### 4.2.14. B16.F10 melanoma model

B16.F10 (5 × 10^5^) cells were suspended in 100 μL PBS and subcutaneously injected into the right flank region of 6–8 weeks old female C57Bl/6j mice (The Jackson Laboratory, Bar Harbor, ME). Established B16.F10 (50 mm^3^) tumors were treated with vehicle (PBS) or STANs (5 μg, every 3 days for 3 injections) intravenously. Tumor volume was measured every other day *via* caliper measurements and volumes were calculated using:*V*_tumor_ = 0.5 × *L* × *W*^2^

In which, *V*_tumor_ is the tumor volume, *L* is the tumor length, and *W* is the tumor width. Mice were euthanized *via* CO^2^ asphyxiation when tumor volume exceeded 1500 mm^3^. All studies using animals, including surgical and experimental procedures, were completed under an Animal Care Protocol (M2000064) approved by the Vanderbilt University Institutional Animal Care and Use Committee (IACUC). Animal health assessments were completed using standard operating procedures approved by Vanderbilt University IACUC.

#### 4.2.15. Statistical analyses

All statistical analyses were performed using GraphPad Prism software version 9.5.1. Error bars on graphs denote standard deviation. All graphs depict mean ± SEM unless otherwise indicated. All statistical analyses presented are one-way ANOVAs with Tukey's multiple comparisons unless otherwise indicated. *P*-Values are depicted as: *P < 0.05, **P < 0.01, ***P < 0.001, ****P < 0.0001.

## Author contributions

H. M. P., P. T. S., and J. T. W. conceived of and designed the experiments. H. M. P. and P. T. S. equally performed a majority of the experiments and data analysis. B. K. assisted with tumor therapy studies. J. J. B. and L. E. P. helped establish FNP methodology using the PEG-DB copolymer system. M. H. A. and L. H. assisted in polymer synthesis, pH response and membrane lysis investigations, and nanopartile loading studies. E. W. R. performed the cryoTEM experiments. S. A. performed the SAXS analysis. E. A. S. guided the overall project direction and provided manuscript feedback. H. M. P., P. T. S., and J. T. W. wrote the manuscript.

## Conflicts of interest

J. T. W. is an inventor on US Patent 10696985 “Reversibly crosslinked endosomolytic polymer vesicles for cytosolic drug delivery” and on US Patent application PCT/US2019/058945 “Graft copolymers, methods of forming graft copolymers, and methods of use thereof,” which both describe drug delivery technologies that have been used for STING agonist delivery.

## Supplementary Material

NR-015-D3NR02874G-s001
