# Supplementary material for: Engineering endosomolytic nanocarriers of diverse morphologies using confined impingement jet mixing
Source: Nanoscale. 2023 Sep 19;15(39):16016–29. doi: 10.1039/d3nr02874g (PMC10568979; doi:10.1039/d3nr02874g)
Supplement: NR-015-D3NR02874G-s001 [file NR-015-D3NR02874G-s001.pdf]

Electronic Supplementary Material (ESI) for Nanoscale.
This journal is © The Royal Society of Chemistry 2023

Electronic Supplementary Information

Engineering endosomolytic nanocarriers of diverse morphologies using confined impingement jet mixing

Hayden M. Pagendarm[1,^], *Payton T. Stone*[2,^], *Blaise Kimmel*[2], *Jessalyn J. Baljon*[1], *Mina H. Aziz*[3,4], *Lucinda E. Pastora*[2], *Lauren Hubert*[5], *Eric W. Roth*[6], *Sultan Almunif*[7], *Evan A. Scott*[7-11], *John T. Wilson*[1,2,12-16*]

[1]Department of Biomedical Engineering, Vanderbilt University, Nashville, TN 37235, USA
[2]Department of Chemical and Biomolecular Engineering, Vanderbilt University, Nashville, TN 37235, USA
[3]Department of Biochemistry, Vanderbilt University, Nashville, TN 37235, USA
[4]Department of Neuroscience, Vanderbilt University, Nashville, TN 37235, USA
[5]Department of Chemical Engineering, The University of Rhode Island, Kingston, RI 02881, USA
[6]NU*ANCE* BioCryo, Northwestern University, Evanston, IL 60208, USA
[7]Department of Biomedical Engineering, Northwestern University, Evanston, IL 60208, USA
[8]Interdisciplinary Biological Sciences, Northwestern University, Evanston, IL 60208, USA
[9]Chemistry of Life Processes Institute, Northwestern University, Evanston, IL 60208, USA
[10]Simpson Querrey Institute, Northwestern University, Chicago, IL 60611, USA
[11]Robert H. Lurie Comprehensive Cancer Center, Northwestern University, Chicago, IL 60611, USA
[12]Vanderbilt Institute for Infection, Immunology, and Inflammation, Vanderbilt University Medical Center, Nashville, TN 37232, USA
[13]Vanderbilt Center for Immunobiology, Vanderbilt University Medical Center, Nashville, TN 37232, USA
[14]Vanderbilt Institute of Chemical Biology, Vanderbilt University, Nashville, TN, 37232
[15]Vanderbilt Institute of Nanoscale Science and Engineering, Vanderbilt University, Nashville, TN 37232, USA
[16]Vanderbilt-Ingram Cancer Center, Vanderbilt University Medical Center, Nashville, TN 37232, USA

^These authors contributed equally to this work

*Corresponding Author: john.t.wilson@vanderbilt.edu

A

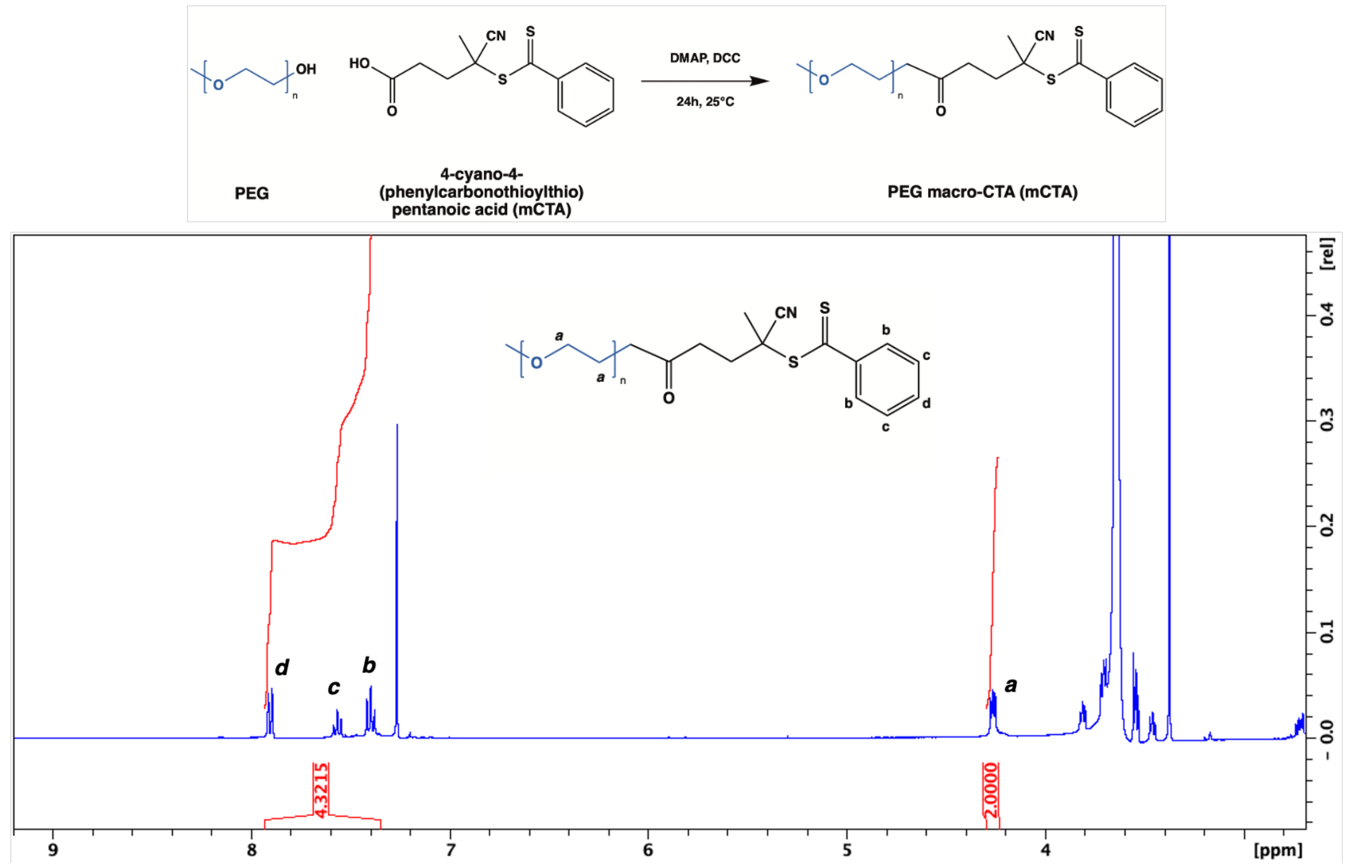

B

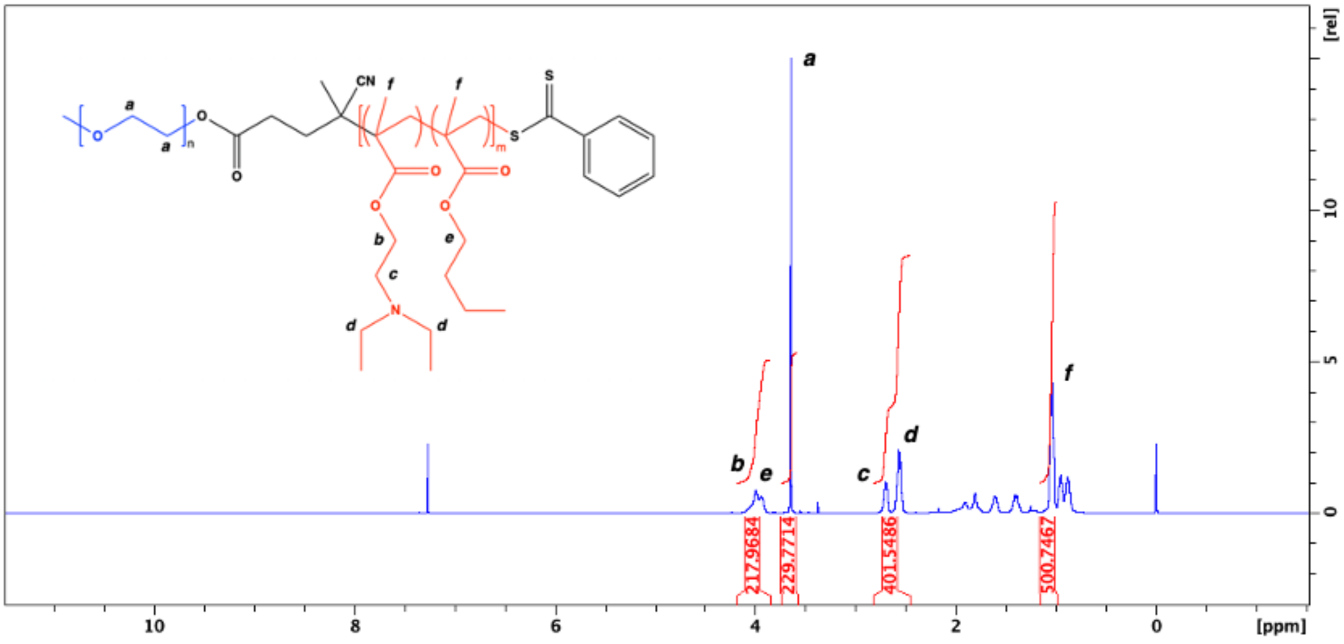

Figure S1. Macro CTA Synthesis and Polymer characterization. (A) Synthesis and representative ^{1}H-NMR analysis ($CDCl_3$) of PEG macro-CTA, and **(B)** representative ^{1}H-NMR analysis ($CDCl_3$) of [PEG]-*bl*-[DEAEMA-*co*-BMA] diblock copolymer.

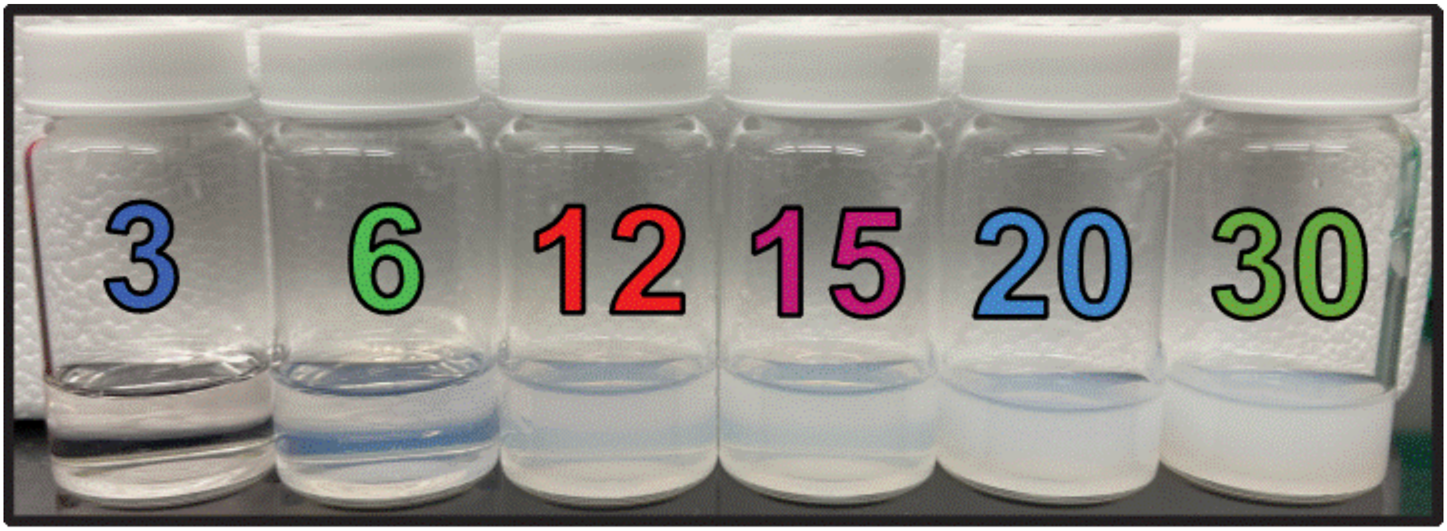

Figure S2. Images of Nanoparticle Suspensions. Representative images of nanocarrier samples suspended in water captured directly after FNP fabrication and purification.

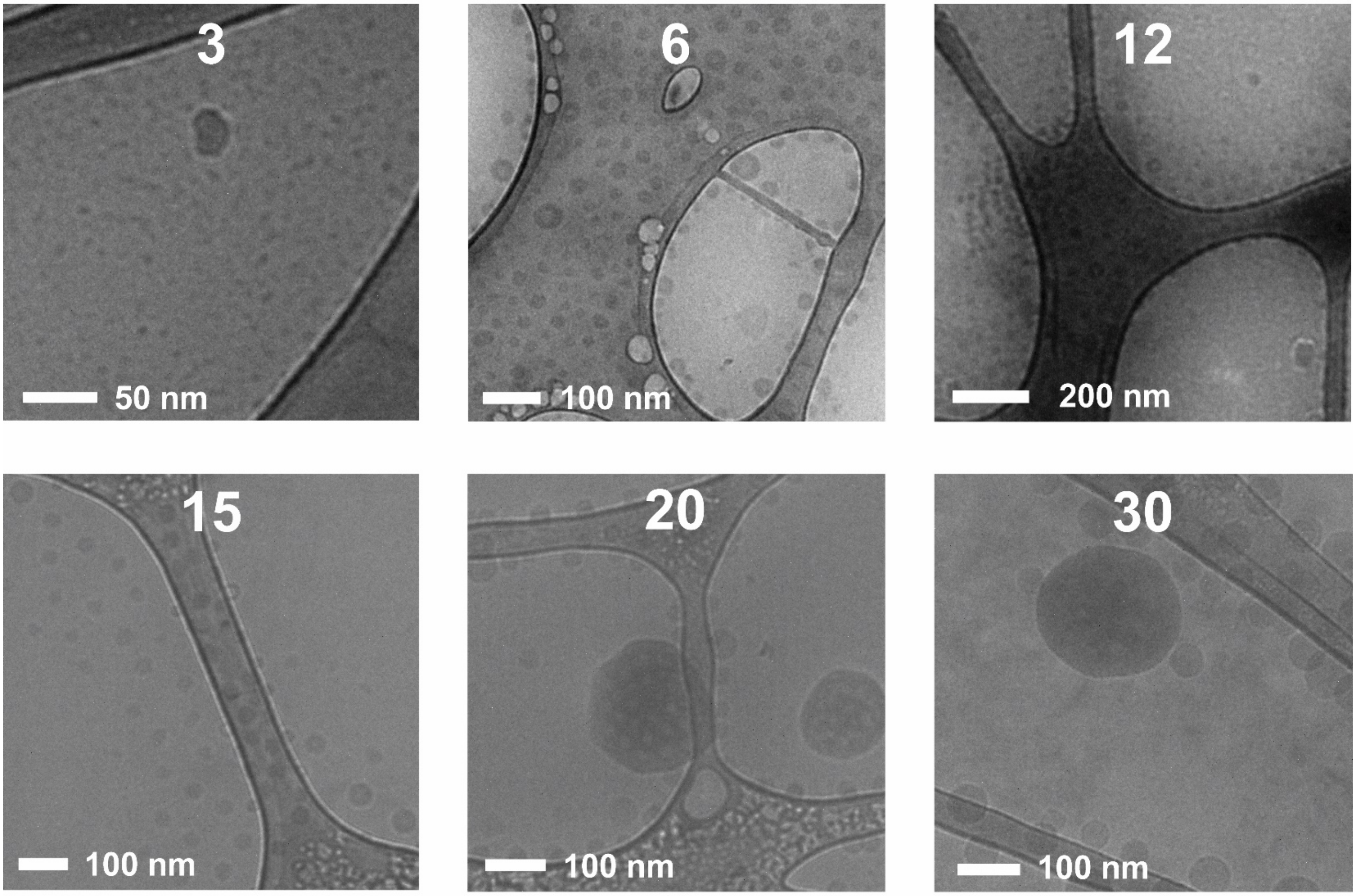

Figure S3. Cryogenic Electron Microscopy (cryoEM) of 1 Impingement Samples. Representative CryoEM images of polymeric nanocarriers fabricated with 1 impingement.

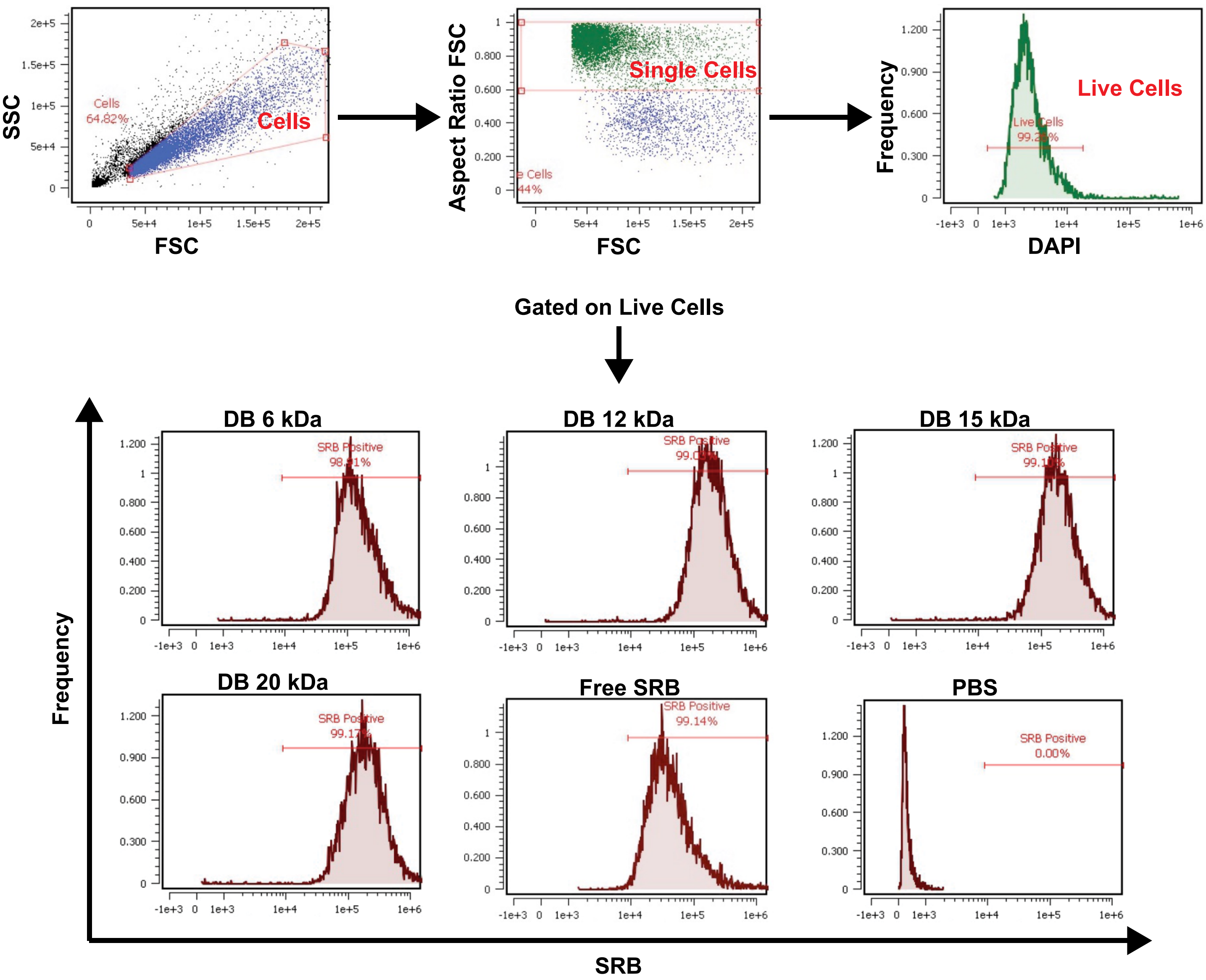

Figure S4. Gating Strategy for Flow Cytometric Analysis of Cellular SRB Uptake. Representative plots from mice treated with nanocarrier formulations encapsulating SRB. Events were first gated on cells (FSC vs. SSC) and then gated on single cells (FSC vs Aspect Ratio FSC). The Aspect Ratio FSC parameter enables the removal of doublets by positively selecting cells with sufficiently high (> 0.6) aspect ratios, calculated as width/length. Events with aspect ratios < 0.6 tend to reflect elongated cells, aggregates, or images capturing more than a single cell in one frame. Single cells were subsequently gated to be DAPI$^-$ to identify live cells, as DAPI uptake indicates cell death. Within the Live Cells population, the median fluorescence intensity (MFI) of the SRB channel was plotted to reflect the relative cellular uptake of SRB by each formulation and control. Representative histograms are displayed above.

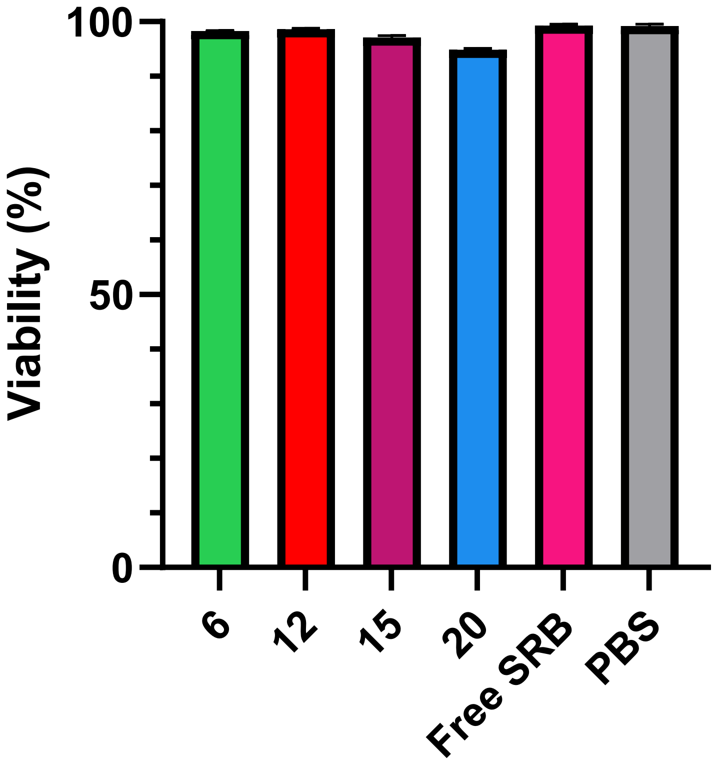

Figure S5. Viability of MDA-MB-231 Cells Following Treatment with SRB-Loaded Nanocarriers. Viability of MDA-MB-231 cells following treatment with 20 μg/mL SRB within indicated nanocarriers. From “Live Cells” gate described in Fig. S4.

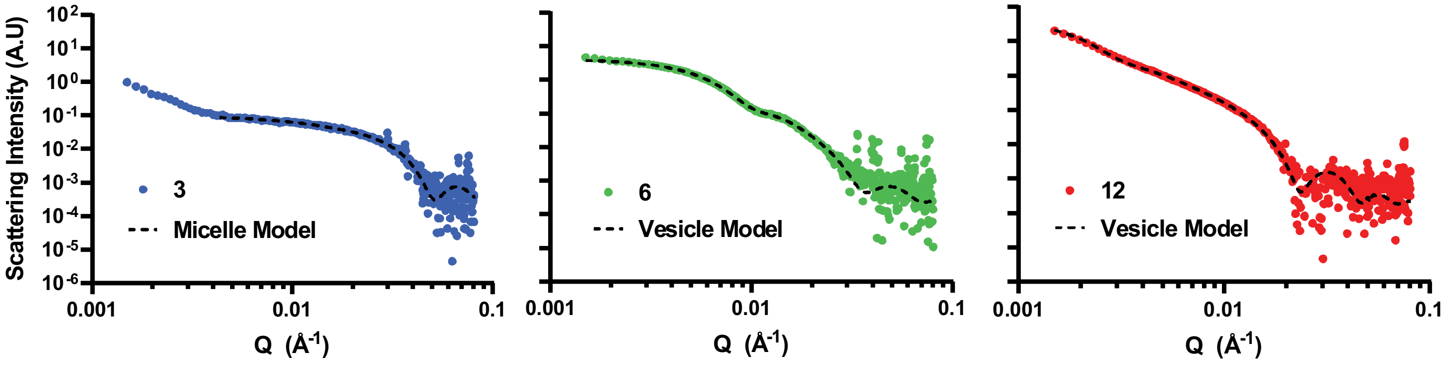

Figure S6. Small-Angle X-ray Scattering (SAXS) Analysis. X-ray scattering curves of DB3kDa (*left*), DB6kDa (*center*), and DB12kDa (*right*) nanocarriers fitted to models of micelles, vesicles, and vesicles, respectively, using the SasView 5.0.5 software package.
